# Improving pan-genome annotation using whole genome multiple alignment

**DOI:** 10.1186/1471-2105-12-272

**Published:** 2011-06-30

**Authors:** Samuel V Angiuoli, Julie C Dunning Hotopp, Steven L Salzberg, Hervé Tettelin

**Affiliations:** 1 Center for Bioinformatics and Computational Biology, University of Maryland, College Park, MD 20742, USA; 2 Institute for Genome Sciences (IGS), University of Maryland Baltimore, Baltimore, Maryland 21201, USA

## Abstract

**Background:**

Rapid annotation and comparisons of genomes from multiple isolates (pan-genomes) is becoming commonplace due to advances in sequencing technology. Genome annotations can contain inconsistencies and errors that hinder comparative analysis even within a single species. Tools are needed to compare and improve annotation quality across sets of closely related genomes.

**Results:**

We introduce a new tool, Mugsy-Annotator, that identifies orthologs and evaluates annotation quality in prokaryotic genomes using whole genome multiple alignment. Mugsy-Annotator identifies anomalies in annotated gene structures, including inconsistently located translation initiation sites and disrupted genes due to draft genome sequencing or pseudogenes. An evaluation of species pan-genomes using the tool indicates that such anomalies are common, especially at translation initiation sites. Mugsy-Annotator reports alternate annotations that improve consistency and are candidates for further review.

**Conclusions:**

Whole genome multiple alignment can be used to efficiently identify orthologs and annotation problem areas in a bacterial pan-genome. Comparisons of annotated gene structures within a species may show more variation than is actually present in the genome, indicating errors in genome annotation. Our new tool Mugsy-Annotator assists re-annotation efforts by highlighting edits that improve annotation consistency.

## Background

Advances in genome sequencing technologies have enabled sequencing of thousands of microbial genomes [1]. Often a single reference genome is insufficient to describe the genetic diversity of a species, leading to sequencing of many closely related isolates and subsequent comparative analysis. To aid in the analysis, an annotation process is typically performed using computational methods that include prediction of genes and their functions. Gene prediction algorithms for prokaryotes have been shown to perform well with relatively low error rates [2-4]. Limitations of gene prediction include accurate identification of the translation initiation start (TIS) sites and pseudogenes, and over-annotation in GC-rich genomes [5]. Specialized tools have addressed these issues, such as for improved TIS prediction [6]. In addition, post-processing can be used to identify annotation anomalies, as in GenePrimp [7].

While there are several tools for gene prediction of single genomes, relatively few tools exist to deal specifically with the simultaneous annotation of large numbers of nearly identical sequenced isolates, such as a species pan-genome. Also, despite low error rates in gene calling, the accumulation of errors across many genomes can cause problems for comparative analysis, such as identification of the conserved core genome [8]. Additionally, as genomes are sequenced and annotated by diverse scientists, annotations can vary due to choice of gene prediction algorithms or annotation procedures [7,9-11].

Re-annotation efforts have been used to standardize annotation across many genomes to a single protocol [12]. This approach is particularly useful for updating out dated annotation with the latest available evidence. A challenge for standardization efforts is combining automated re-annotation while preserving curated edits, which may include corrections of gene prediction errors. This process requires integration of both manually curated structures and *ab-initio *gene predictions.

Comparative analysis of closely related sequences forms the basis of many annotation approaches [13]. Reference-based approaches that map annotation onto new genomes using a reference [14] are particularly well-suited to annotation within a species where many genes are expected to be identical in each sequenced isolate. For some species, the use of a single reference genome can be limiting and as a result, researchers often need to integrate annotations from multiple sources. Whole genome multiple alignment is well suited for comparative analysis of closely related genomes, including in a reference independent manner [15-17]. The multiple whole genome alignment tool, progressive Mauve [15], provides a feature for reporting orthologous genes as indicated by the alignment allowing for comparisons of genes across genomes. While fully automated approaches for comparison and annotation are of heightened interest as genome sequencing throughput has increased, the need for combining manual, expert curation with high-throughput automated approaches has been recognized [18].

In this study, we introduce a new tool, Mugsy-Annotator, that uses whole genome multiple alignment for two objectives: 1) identifying orthologs and 2) evaluating the quality of annotated gene structures in prokaryotic genomes. The method is effective for identification of aligned genes, such as orthologs, whose genomic position is conserved. The method also provides the foundation for comparing gene structures to identify annotation anomalies, including inconsistently annotated translation initiation sites (TIS), missing annotations, and disrupted genes due to sequencing and assembly errors, or pseudogenes, including frameshifted genes. Finally, Mugsy-Annotator identifies alternative annotations that can resolve anomalies and improve annotation consistency. The tool is freely available at http://mugsy.sf.net.

## Methods

**Figure 1:**
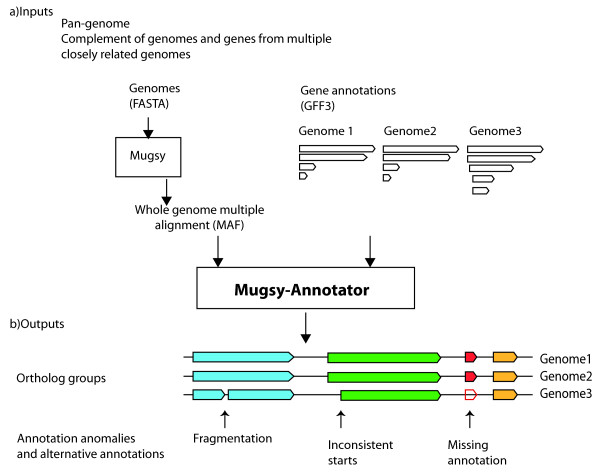
**Identifying orthologs and comparing gene structures in a pan-genome using whole genome multiple alignments**. The input is provided as a set of genomic sequences (FASTA format) and gene annotations (GFF3 format). Whole genome multiple alignments (top left) are first calculated using Mugsy [16]. Mugsy-Annotator then builds groups of orthologous gene structures that are conserved in sequence and genomic context according to the alignment. The alignment also indicates the location of each predicted translation initiation start and stop across the genomes, allowing for identification of annotation anomalies or missing annotations.

The method consists of three primary steps, (1) aligning multiple whole genomes, (2) mapping orthologs among the genomes, and (3) identifying annotation anomalies (Figure 1). Two types of input files are required: genome sequences in FASTA format, and annotated gene structures (CDS features) in Genbank or GFF3 format. It is expected that a gene prediction algorithm has been run on all input genomes. For step 1, we generate reference-independent whole genome multiple alignments (WGA) using Mugsy [16]. The alignments generated by Mugsy are restricted to a single region per genome and used by Mugsy-Annotator to define orthologous relationships between sequences. Mugsy outputs alignments in Multiple Alignment Format (MAF) that are passed to Mugsy-Annotator along with the genome annotations needed to complete steps 2 and 3. The genomic coordinates and alignment string of each aligned interval are extracted from the MAF files and stored in an interval tree [19] to provide fast querying of genomic intervals. The start and end coordinates of each gene are also extracted as intervals from the annotation files and stored in the interval tree. The interval tree is then queried by Mugsy-Annotator to build groups of orthologs and identify anomalies in gene boundaries. Although we utilize Mugsy for whole genome multiple alignment, Mugsy-Annotator accepts MAF files as input and other whole genome alignments tools can be used instead of Mugsy as long as the input is properly formatted.

### Identification of orthologs

Sets of orthologs are determined by retrieving genes whose intervals are aligned via whole genome alignment (WGA). First, the input genes are sorted on length. The longest gene remaining in the input set, termed the query gene, is removed from the input and used to define a new ortholog group. Genes from other species that align to the query gene in the WGA are added to the ortholog group and removed from the input set. This ensures genes are placed in exactly one group. A configurable coverage cutoff can limit consideration to alignments that span a minimum percentage of the query gene and other matching genes. In this study, we set these length cutoffs to 50%. The procedure continues in a greedy fashion using the longest remaining gene to seed new groups (or clusters) until no genes are remaining. Query genes with no overlapping genes above the cutoffs are reported as singleton groups. Using this method, the query gene in each ortholog group is at least as long as any other gene in the cluster and may span multiple adjacent genes in other genomes. This allows our method to identify apparent fragmented genes within a single region in cases where fragments are conserved in order with respect to an alignment to a single continuous gene.

Mugsy-Annotator expects one segment per organism in the whole genome alignment. In cases of segmental duplications, Mugsy-Annotator will report separate ortholog groups for each copy only if whole genome alignment identifies orthologous copies in other genomes, using both sequence identity and position in determining which copy is orthologous. In other cases, Mugsy-annotator does not recognize duplications and will report them as singletons clusters. The Mugsy whole genome aligner does identify additional duplications but this information is not currently interpreted by Mugsy-Annotator.

To generate OrthoMCL clusters for comparison [20], we performed an all-against-all BLASTP searches of conceptual translations of the gene predictions. BLASTP alignments with e-value < 10^-5 ^were used as input to OrthoMCL v1.4 to predict groups of orthologs.

### Identification of annotation inconsistencies

**Figure 2:**
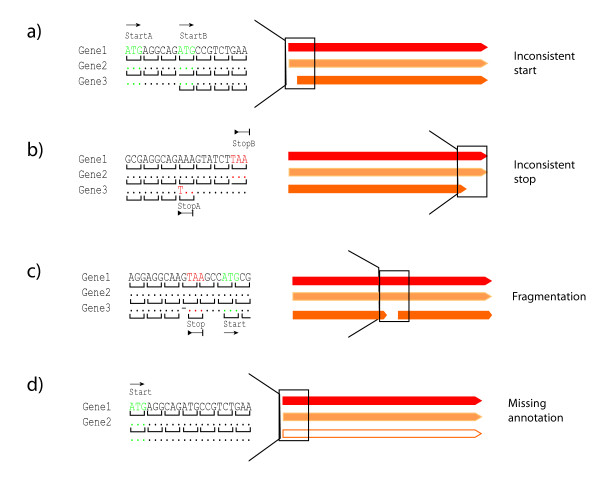
**Annotation anomalies identified by Mugsy-Annotator**. Four classes of anomalies are shown (a-d). On the right, examples of aligned genes are drawn with the boxed region indicating the location of the anomaly. On the left, a multiple alignment is depicted across the highlighted region with sequence identity indicated by dots. In (c), a gap indicated by a dash introduces a shift in reading frame that results in use of a termination codon that is inconsistent with the annotations in the other genomes. Translation initiation sites are marked as "start" and termination codons are marked as "stop" with an arrow indicating the direction of translation.

Mugsy-Annotator produces a report of the annotation consistency for each ortholog set. To classify annotation consistency for each ortholog set, we examine the location of the annotated start and stop codons for each gene in the multiple alignment. If all annotated start and stop codons are in the same location, the ortholog set is consistently annotated and we identify no inconsistencies. Otherwise, we classify the ortholog set into one or more classes: inconsistent starts, inconsistent stops, and multiple gene fragments. If the stop codon locations are the same for all annotated genes but the TIS differ, we classify the set as inconsistent starts (Figure 2a). If the start codon locations are the same for all genes but the stop codon locations differ, we classify the set as inconsistent stops (Figure 2b). If both start and end locations differ for some members of the group, we classify the group as a combination class. This class will include genes that overlap in the alignment but in different reading frames or strands. Aligned gene sets with multiple annotated genes in the same genome are classified as multiple gene fragments (Figure 2c).

### Alternative annotations

Mugsy-Annotator suggests edits that can resolve anomalies and improve the consistency of each aligned gene set. To determine the possible edits, start and stop codons pairs from each aligned set are checked against the WGA to determine if the aligned positions correspond to ORFs with a valid translation start and stop site (NCBI translation table 11) in each of the other aligned genomes. In cases where the region already contains gene predictions, only alternatives that are greater than a specified percentage (50% by default) of the annotated length are considered.

The procedure will also identify aligned gene sets with multiple gene fragments that can be merged into a single spanning gene by introducing a point mutation or frameshift into the annotation. If the aligned regions contain gaps, Mugsy-Annotator attempts to introduce a frameshift to create a valid ORF joining the start and stop codon pair. Start and stop codon pairs are then displayed ordered by the number of valid ORFs and their length, although this sort order is configurable. This procedure will also identify possible missing genes in regions of the genome that are aligned to other annotated genes (Figure 2d). To be considered a missing annotation, there must be no overlapping gene predictions in the aligned interval.

### Data sets

The *Neisseria meningitidis (Nmen) *dataset of 20 genomes was the same as used in [21]. Two versions of the annotation were available, *Nmen *verA and *Nmen *verB. *Nmen *verA contained 13 genomes that had been annotated using one of two automated pipelines prior to any manual review. Unless noted, the annotation anomalies identified in this study used the *Nmen *verB annotations, which had undergone limited manual review. The remaining species pan-genomes used in this study (listed in Additional file 1, Table S1) were downloaded from the Refseq database [22]. MUMi [23] distance measurements were calculated for each pair of sequences with a named species.

## Results

### Mugsy-Annotator for finding orthologs

Mugsy-Annotator uses whole genome alignment (WGA) calculated by Mugsy [16] to identify conserved genes in a set of genomes (Figure 1). In cases where the alignment represents orthologous regions, these aligned genes correspond to orthologs; i.e., genes descended from the same ancestral sequence. WGA aids in distinguishing orthologs from paralogs by identifying regions that are syntenic and conserved in both sequence and chromosomal position. By aligning genomic DNA, WGA can also identify erroneous gene predictions in a reading frame that produce a nonsense translation and escape detection by similarity methods that rely on conceptual translations, such as BLASTP. On the other hand, by relying on DNA alignment, Mugsy-Annotator might miss sequence conservation between genes that is only detectable at the protein level.

To evaluate the properties of WGA as a method for ortholog identification, we compared the groups of orthologs for 20 *Neisseria meningitidis (Nmen) *genomes reported by Mugsy-Annotator and a popular BLAST-based clustering method, OrthoMCL [21]. OrthoMCL performs a clustering of Reciprocal Best BLAST (RBB) matches between conceptual translations of genes to identify orthologs. In *Nmen*, Mugsy-Annotator identified 2,440 ortholog groups compared to 2,320 reported by OrthoMCL. The Mugsy-Annotator groups include nearly all the genes included in RBB matches used by OrthoMCL (38,905 of 39,593 total, 98%).

**Figure 3:**
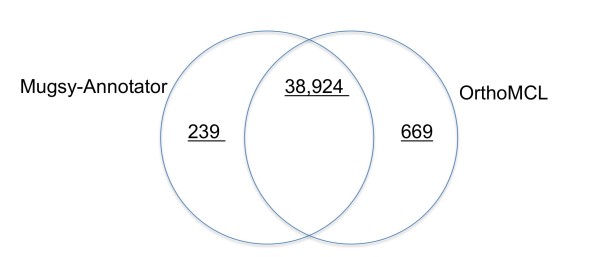
**Comparison of genes reported in orthology groups from Mugsy-Annotator and OrthoMCL**. The intersection between Mugsy-Annotator and OrthoMCL reports the number of genes reported in ortholog groups by both methods. The remainder for Mugsy-Annotator and OrthoMCL reports the number of genes classified in ortholog groups by one of the methods only.

Both methods reported genes missing from groups reported by the other method, totaling 239 and 669 genes reported by Mugsy-Annotator and OrthoMCL exclusively (Figure 3). Many of the genes reported exclusively by one method appear to be paralogs based on intra-genome BLASTP matches (40% and 66% reported exclusively by Mugsy-Annotator and OrthoMCL methods respectively) or have functional names that indicate transposases (33% and 23% for WGA and RBB respectively) or hypothetical proteins (34% and 31% for Mugsy-Annotator and OrthoMCL respectively).

The comparison of our WGA based method and a BLAST based method is illustrative of the differences between the methods in grouping paralogs. Clustering of RBB matches can collapse orthologs and paralogs into a single group. In the *Nmen *comparison, OrthoMCL reports 310 groups with multiple genes per genome that align to each other via BLASTP, indicating paralogs in a single group. Mugsy-Annotator will sometimes report groups with more than one gene per genome (Figure 2c, "Fragmentation"), but rather than paralogs, these groups represent fragmented genes due to draft genome sequencing (gaps or sequencing errors) or potential pseudogenes. As described in Methods, our tool, by utilizing WGA, incorporates genome context and synteny in determining matches and builds groups that are restricted to a single gene copy per genome, thus avoiding the grouping of orthologs and paralogs together. Identifying orthologs separately is needed for phylogenetic analysis of gene families that rely on orthologs, comparison of upstream regulatory regions, and examination of segmental duplications, where each duplicated copy has a distinct genomic context. In other cases, grouping of paralogs and orthologs together is desirable and as such our WGA-based method is expected to be complementary to BLAST based clustering methods for identifying gene families.

For genes grouped exclusively by Mugsy-Annotator, 23 have no reported intra-species BLASTP matches to other genes in *Nmen*, and include annotations that appear to be in an incorrect ORF (Additional file 2, Table S2). Although we found this class of anomaly to be rare in our evaluation, Mugsy-Annotator, by using WGA, is able to identify orthologs to such regions that lack BLAST matches within the dataset and may have a nonsense conceptual translation. An additional 68 genes (28%) reported exclusively by Mugsy-Annotator are adjacent to contig boundaries and may be truncated gene predictions that escape detection by BLAST.

Our WGA method is computationally efficient and has a significant runtime performance advantage over BLAST. The comparison of 20 *Nmen *genomes runs on a single CPU in ~4 h (~2 h for WGA with Mugsy and ~2 h for comparing annotations with Mugsy-Annotator). By comparison, the exhaustive all-against-all BLAST of predicted proteins needed for OrthoMCL consumed ~32 CPU hours and was run on a compute cluster to obtain a faster runtime. In addition, BLAST-based methods that rely on searches of conceptual translation may require additional search of the genomic DNA, such as with BLASTx, to confirm gene presence and avoid mis-prediction of paralogs as orthologs.

### Missing annotations

**Figure 4:**
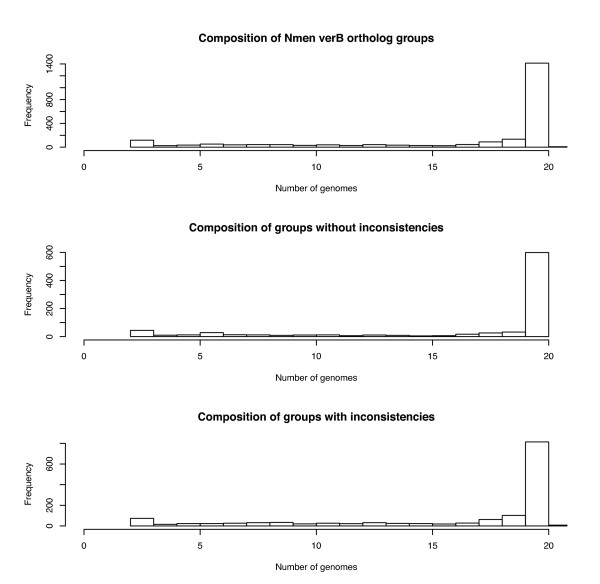
**Distribution of the number of genomes in ortholog groups identified by Mugsy-Annotator for 20 *Nmen *genomes**. The number of genomes per orthology groups are provided for all orthology groups (top), consistently annotated groups only (middle), and exclusively groups with annotation inconsistencies (bottom).

Mugsy-Annotator can be used to identify missing annotations and putative genes by looking for regions of the alignment with a prediction in some genomes but not others (Figure 2d, "Missing annotation"). These missing annotations can arise from use of varying gene prediction tools and uncertainty in gene calling procedures, especially for short genes [24]. In our study of 20 *Nmen *strains, a majority of the aligned gene sets contain one annotated region from each of the genomes (Figure 4) and missing gene predictions were rare, totaling only 50 genes missing in alignments containing 18 or more genomes (Additional file 3, Table S3).

Mugsy-Annotator identifies missing annotations if DNA corresponding to a putative gene is an ORF that is conserved across genomes. However, it does not provide additional evidence to determine if a gene prediction is missing in some genomes (false negative) or there is an overcall in the other aligned genomes (false positive). Our methodology relies on sequence conservation between the input genomes, which by itself is insufficient to distinguish between these due to the short phylogenetic distance and high similarity of the genomes. Examination of additional evidence (eg. HMM or BLAST searches) or experimental validation is required to differentiate between these cases.

### Identifying and resolving annotation anomalies

To aid in re-annotation efforts, Mugsy-Annotator identifies likely annotation problem areas and suggests alternative genes based on the whole genome multiple alignment. To find such problem areas, Mugsy-Annotator first examines each of the aligned gene sets for inconsistencies in annotated gene boundaries amongst members of the set (Figure 2). The reported anomalies include inconsistently located TIS, disrupted genes, or alternative ORFs. Mugsy-Annotator then generates a report for each aligned gene set that describes the inconsistency and possible resolutions. A browser of the annotations overlaid on the whole genome multiple alignment is also provided (Additional file 4, Figure S1).

**Figure 5:**
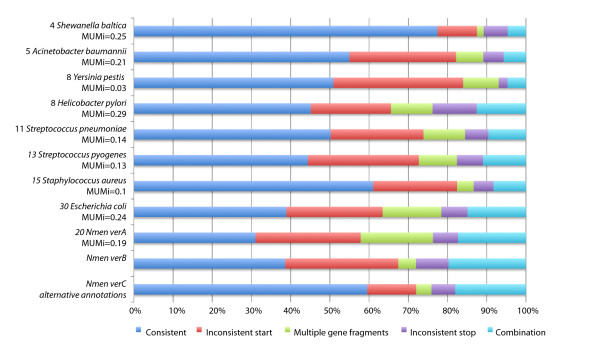
**Consistency of annotated gene structures in several species pan-genomes as reported by Mugsy-Annotator**. Each row provides the fraction of aligned gene sets in each class of anomaly and groups with no identified inconsistencies (blue). The number of genomes compared and their average MUMi similarity [23] distance is also provided, ranging from zero for most similar to 1, least similar. The bottom three rows describe three versions of annotations from the case study of *Neisseria meningitidis (Nmen) *. The last version (*Nmen *verC) demonstrates improvements in consistency using alternative annotations suggested by Mugsy-Annotator.

To demonstrate the tool, we ran Mugsy-Annotator on nine bacterial species, all of which have multiple strains with complete genomes available (Figure 5). The output indicates many inconsistencies in annotated gene structures, with inconsistent TIS locations as the most commonly identified anomaly. While the inconsistencies may indicate errors in the annotated gene structures in one or more of the genomes, the results are not surprising as the sequencing coverage, date of annotation, and annotation protocols vary. The presence of annotation errors in public repositories has been widely recognized [25-27] leading to a number of re-annotation efforts for genomes in a single species [28,29].

**Figure 6:**
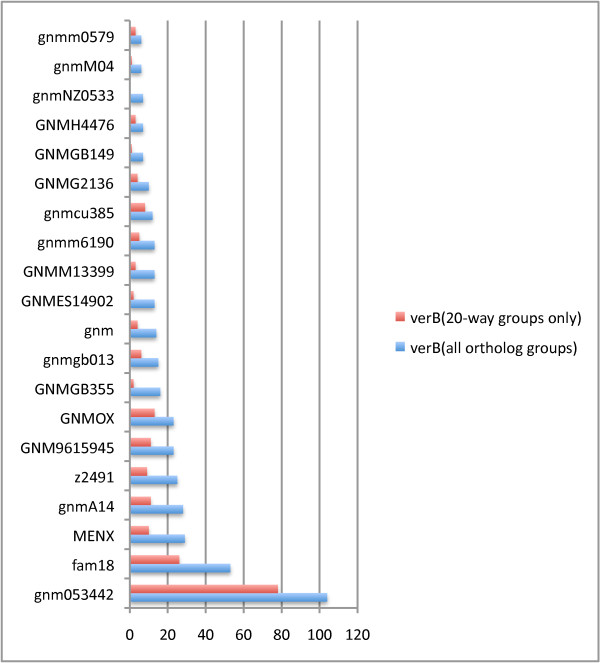
**Annotation anomalies caused by a single genome**. Each row provides a count of ortholog groups where the named genome is inconsistent with the remaining genomes in the group. In these cases, the annotated translation initiation site in the named genome in *Nmen *verB did not match any of the other annotated gene structures in the ortholog groups.

As a case study, we evaluated the Mugsy-Annotator report for the dataset of 20 *Nmen *genomes. Inconsistent TIS are the most commonly detected anomaly in *Nmen *with 30% of aligned gene sets containing more than one annotated TIS. Due to lack of precision in TIS prediction, we expect the number of TIS inconsistencies to increase as the number of genomes increases, especially since our method marks a group as inconsistent even if the annotation error is limited to a single genome. To see how overall consistency is affected by any single genome, Mugsy-Annotator reports the number of times a single genome is inconsistent in comparison to the set. An examination of the *Nmen *genomes shows that certain subsets of genomes have better internal consistency. In 27% of groups with TIS inconsistencies, an alternative annotation in a single genome will resolve the inconsistencies for the group (Figure 6). Although some of the *Nmen *genomes contributed to more annotation inconsistencies than others, all of the genomes contributed to inconsistencies in at least one group.

**Figure 7:**
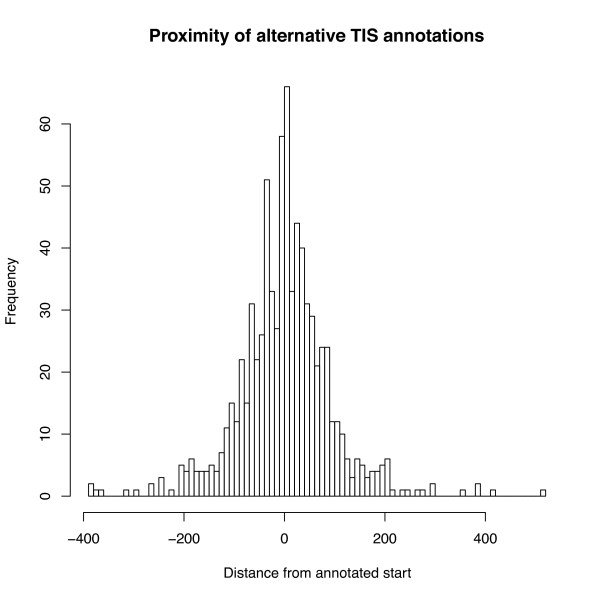
**Distance of alternative TIS from the annotated site**. Distance between the annotated translation initiation site and the most consistent translation initiation site reported by Mugsy-Annotator.

Mugsy-Annotator suggests alternative gene structures that improve annotation consistency. In *Nmen *core gene groups containing all genomes, 55% (400/725) of groups with inconsistent TIS can be resolved by an alternative annotation that is conserved across all the aligned genomes. In 50% of these cases, the alternative start site is upstream of the existing annotation, resulting in longer annotations. In the remaining cases, the most consistent TIS location results in a shorter gene in at least one genome. A majority of the alternative TIS locations are in the same coding frame and within 42 bp of the annotated TIS (Figure 7), indicating that annotation protocols have chosen inconsistently between sites that are nearby along the genome. Adjusting the TIS can result in an overlap with an adjacent gene. To help avoid mis-annotation of overlapping genes [30], Mugsy-Annotator flags edits that would result in an overlap with an adjacent gene. In alternative annotations of *Nmen *groups, 15% (63/400) introduce overlap with adjacent annotations indicating further evaluation is needed to determine the correct annotation.

When a single gene in one genome is aligned to multiple genes in other genomes, Mugsy-Annotator calls this an anomaly (Figure 2c, "Fragmentation"). These apparent gene fragments can arise from sequencing and assembly errors; from interesting novel gene fusions; or from pseudogenes, in which frameshifts or in-frame stop codons can split an open reading frame into multiple gene-like fragments. In our case study of *Nmen*, draft genome sequencing appears to contribute to a vast majority of occurrences of this anomaly (Additional file 5, Table S4), although the tool has also aided in the identification of several novel gene fusions that are not fixed in the population. To aid in classifying this anomaly further, Mugsy-Annotator reports whether or not frameshifts can extend the interrupted gene fragments to match a longer annotated gene. Amongst the aligned gene sets containing all 20 *Nmen *genomes, Mugsy-Annotator found 48 cases where a single previously un-annotated frameshift would resolve the anomaly and result in a consistently annotated set (Additional file 6, Table S5). In many other cases, some of the genes can be extended with a frameshift but other anomalies remain in the group. Additional review would be needed to further classify these anomalies. To aid with this, Mugsy-Annotator provides a view of the current annotations and reading frames overlaid on the whole genome multiple alignment (Additional file 4, Figure S1).

In the *Nmen *study, Mugsy-Annotator suggests alternative annotations that can improve consistency in up to 57% of ortholog groups. Although the alternatives improve consistency, in most cases an examination of additional evidence is required to ensure that edits improve quality. In this case study, the variability of the annotation is partly due to the multitude of sources and sequencing strategies. The *Nmen *genomes are available in varying stages of sequence quality including 9 draft and 11 complete genomes and the annotation evaluated came from a total of 5 laboratories using varying gene prediction protocols and levels of manual curation. To better accommodate draft genomes, the gene prediction procedure used in some of the *Nmen *genomes allows for partial open reading frames that terminate or initiate outside of a contig boundary. Mugsy-Annotator flags anomalies that are caused by these partial genes adjacent to contig boundaries. In *Nmen*, such cases contributed to ~9% of start and stop site inconsistencies and at least 67% of all of the multiple gene fragment anomalies (Additional file 5, Table S4). Annotation anomalies due to draft genome assemblies will continue to be an issue in multi-genome analysis as current generation sequencing technologies have prompted an explosion in the number of draft genomes.

To demonstrate annotation improvements using Mugsy-Annotator, we scored annotation consistency in three versions of annotation for *Nmen*. An initial version of the *Nmen *annotation (*Nmen *verA), contained predominantly automated annotation in 13 newly sequenced genomes and curated annotation for 7 complete genomes. *Nmen *verA showed a large number of inconsistencies, encompassing 72% of ortholog groups (Figure 5). As part of the study in [21], limited manual curation was performed and resulted in annotation of frameshifts and removal of many short, unsupported hypothetical gene predictions and resulted in the annotations in *Nmen *verB. Although this manual effort was aided by the Mugsy-Annotator report, the curation effort was not meant to be exhaustive and not all reported inconsistencies were examined during the review. Subsequent to this manual effort, Mugsy-Annotator was run again and generated a new set of alternative annotations (*Nmen *verC)suggesting additional improvements were possible. This resulted in consistent annotations in 59% of groups in *Nmen *verC, which was an increase from 28% in *Nmen *verA The improvement in annotation consistency between versions highlights the need for re-annotation and manual review subsequent to automated annotation.

## Discussion

With the growing availability of numerous genomes for many bacterial species, there is an increasing need for tools that can integrate information to produce more consistent, higher quality annotation for each individual genome as well as the pan-genome. Mugsy-Annotator aids in identifying and comparing gene content across a pan-genome, including draft genomes, with an approach that is independent of a reference genome. For re-annotation efforts, Mugsy-Annotator can be used to direct curators to likely errors and make corrections across many genomes simultaneously, rather than one genome at a time. Our case study indicates that comparisons of annotated gene structures show considerable variation that is a consequence of bioinformatics methods rather than true biological differences. Differences appeared greater among translation initiation sites and in regions with poor sequencing coverage.

Mugsy-Annotator is also an efficient, accurate method for finding orthologs within a pan-genome. By using a fast whole genome alignment tool, Mugsy, our method is computationally efficient compared to BLAST-based approaches for finding orthologs. Our approach is also robust for certain types of annotation errors, such as missing annotations or incorrect reading frames. Since our method relies on accurate DNA alignment, it is most useful for closely related genomes that share a high percentage of identical DNA, such as isolates from the same or closely related species. Mugsy-Annotator is currently limited to analysis of annotation that does not contain spliced gene structures.

Mugsy-Annotator currently compares genomes using an existing set of gene predictions. We plan to integrate it into an automated pipeline for *de-novo *annotation of one or more newly sequenced genomes from an existing set of reference genomes. One option for an implementation of this would include integration of sequence conservation defined by WGA into a *de-novo *gene finder as additional evidence supporting the annotation, especially if a well chosen outgroup sequence is provided. Alternatively, a mapping approach, similar to approaches for mapping between two genomes [14], could be used to augment existing gene predictions and transfer names and functional annotations across the new genomes. Since gene prediction runs quickly on bacteria (usually minutes), we expect the speed of such an approach would be limited by the time required to calculate a whole genome multiple alignment.

As many genomes are currently sequenced to draft status and represented in multiple contigs, Mugsy-Annotator can aid in identifying truncated or missing genes that are due to poor sequencing coverage by examining anomalies identified by Mugsy-Annotator that are adjacent to contig boundaries. For investigating anomalies caused by sequencing coverage, at least one relatively complete genome is required in the alignment for the approach to be effective.

Mugsy-Annotator looks for inconsistencies in gene structures to identify likely errors. It is also possible that consistency results from the propagation of an annotation error, especially since it is common to use reference genome annotations when annotating new genomes. In some cases, the annotated gene structures may be consistent but incorrect and Mugsy-Annotator will not identify any anomaly. On the other hand, due to the short evolutionary distance between the genomes under evaluation in our case study, inharmonious gene boundaries in orthologs are expected to indicate an improper gene boundary assignment in at least one genome. Importantly, additional evidence besides the WGA will often be needed to determine the correctness of the annotations including, but not limited to, gene boundaries of more distantly related orthologs, third position compositional bias, predicted ribosomal binding sites, and predicted signal peptides. As such, our tool stops short of determining the correctness of any gene calls, as this is best left to follow-up analysis or experimentation in the laboratory. Yet, our tool is ideally suited to direct the annotation curator towards the regions in most need of attention, and where Mugsy-Annotator suggestions will greatly facilitate rapid improvement of annotation consistency. Such tools are urgently needed in light of the explosion of genomes currently happening as researchers are sequencing hundreds of genomes for many individual species.

## Conclusion

Whole genome multiple alignment can be used to efficiently identify orthologs and annotation problem areas in a bacterial pan-genome. Our new tool Mugsy-Annotator assists re-annotation efforts by highlighting potential annotation errors and suggesting alternative annotations that improve annotation consistency.

## Competing interests

The authors declare that they have no competing interests

## Authors' contributions

SVA devised the method, implemented the tool, and ran evaluations in consultation with JDH, HT, SLS. SVA drafted the manuscript. All authors read and approved the final manuscript.

## Supplementary Material

Additional file 1 **Table S1: List of *Nmen *genomes**. Click here for file

Additional file 2 **Table S2: List of genes found exclusively in whole genome alignment groups**. Click here for file

Additional file 3 **Table S3: List of missing gene annotations in *Nmen***. Click here for file

Additional file 4 **Figure S1: Screenshot of Mugsy-Annotator report of annotation inconsistencies**. An indel in one of the genomes (MENX) introduces a frameshift that results in a premature stop codon location when compared to the other genomes. Mugsy-Annotator identifies this anomaly and reports the location of the frameshift mutation (indicated by the red box). An alternative annotation in MENX that utilizes a +1 frameshift at the location of this single indel results in gene boundaries (TIS and stop codon) that are consistent with the other 19 genomes in the multiple alignment. Click here for file

Additional file 5 **Table S4: List of causes of annotation anomalies**. Click here for file

Additional file 6 **Table S5: List of *Nmen *ortholog clusters from whole genome multiple alignment**. Click here for file
