# Supplementary figures and images for: Improving pan-genome annotation using whole genome multiple alignment
Source: BMC Bioinformatics. 2011 Jun 30;12:272. doi: 10.1186/1471-2105-12-272 (PMC3142524; doi:10.1186/1471-2105-12-272)

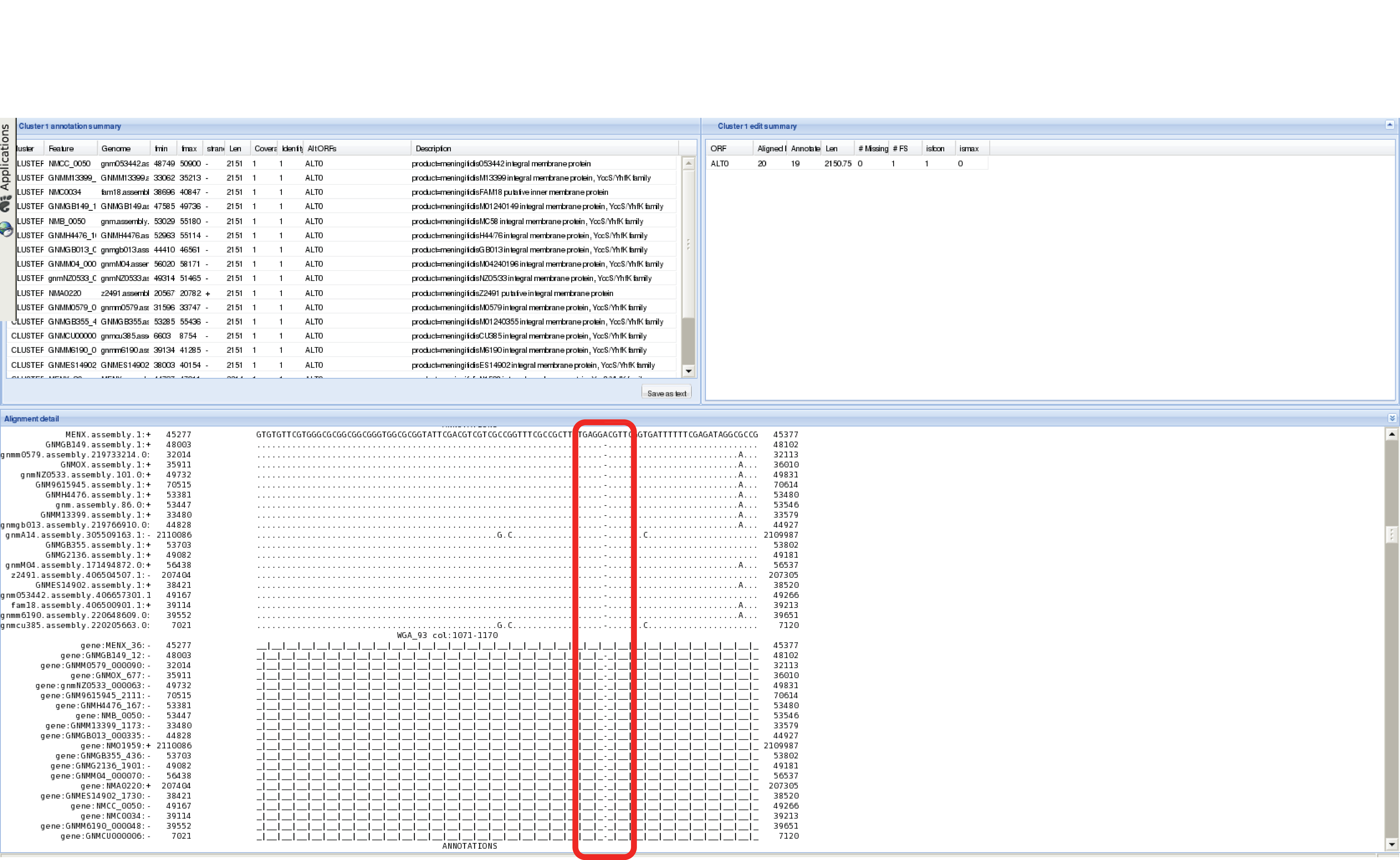

Supplement: Additional file 4 — Figure S1: Screenshot of Mugsy-Annotator report of annotation inconsistencies. An indel in one of the genomes (MENX) introduces a frameshift that results in a premature stop codon location when compared to the other genomes. Mugsy-Annotator identifies this anomaly and reports the location of the frameshift mutation (indicated by the red box). An alternative annotation in MENX that utilizes a +1 frameshift at the location of this single indel results in gene boundaries (TIS and stop codon) that are consistent with the other 19 genomes in the multiple alignment. [file 1471-2105-12-272-S4.PNG]
